# Antibiotics prophylaxis at the time of catheter removal after radical prostatectomy: a systematic review of the literature and meta-analysis

**DOI:** 10.1590/acb390424

**Published:** 2024-02-05

**Authors:** Letícia Lourenço dos Santos, Isabela de Almeida Fraga, Vitor Amaral de Almeida, Andressa Hellen Ribeiro Santos, Isabelle Matos Almeida, Tatiana Roberta Nascimento, Breno Cordeiro Porto, Carlo Camargo Passerotti, Everson Luiz de Almeida Artifon, Jose Pinhata Otoch, José Arnaldo Shiomi da Cruz

**Affiliations:** 1 Universidade Nove de Julho – São Paulo (SP) – Brazil.; 2 Universidade de São Paulo – School of Medicine – Surgical Technique and Experimental Surgery – São Paulo (SP) – Brazil.; 3 Hospital Alemão Oswaldo Cruz – Specialized Center for Urology – São Paulo (SP) – Brazil.

**Keywords:** Prostatectomy, Urinary Tract Infections, Anti-Bacterial Agents, Catheters, Systematic Review, Meta-Analysis

## Abstract

**Purpose:**

To conduct a systematic literature review with meta-analysis to identify whether antibiotic prophylaxis after removal of the indwelling urinary catheter reduces posterior infections.

**Methods:**

A systematic literature review was conducted in the databases PubMed, Embase, Cochrane, Google Scholar, and Latin American and Caribbean Health Sciences Literature, using the keywords “antibiotics” AND “prostatectomy” AND “urinary catheter.”

**Results:**

Three articles were identified having the scope of our review, with 1,040 patients, which were subjected to our meta-analysis revealing a marginally significant decrease in the risk of urinary infection after indwelling urinary catheter removal (odds ratio–OR = 0.51; 95% confidence interval–95%CI 0.27–0.98; p = 0.04; I2 = 0%). No difference was found regarding the presence of bacteriuria (OR = 0.39; 95%CI 0.12–1.24; p = 0.11; I2 = 73%).

**Conclusions:**

In our meta-analysis, there was a significant decrease in urinary tract infection with antibiotic prophylaxis after indwelling urinary catheter removal following radical prostatectomy.

## Introduction

Prostate cancer, a pervasive and potentially lethal disease, ranks as the second leading cause of cancer-related mortality among men^1^. Among the diverse treatment modalities available, radical prostatectomy (RP) emerges as a pivotal intervention. RP patients commonly require short-term indwelling urinary catheters post-surgery to facilitate the primary healing of the vesico-urethral anastomosis.

Despite its life-saving potential, RP introduces unique challenges, notably urinary tract infections (UTIs), which can significantly impact patients’ recovery and overall well-being^1^^,^ 2. The presence of indwelling urinary catheter is a well-known risk factor for UTIs, potentially progressing from asymptomatic bacteriuria to symptomatic UTIs. Managing this condition remains a subject of clinical debate and presents a critical decision point for healthcare providers.

One approach to mitigate UTI risk in this vulnerable patient population involves administering antibiotic prophylaxis at catheter removal^2^. Many clinicians adopt this strategy to preemptively address potential infections. However, the medical community lacks consensus on whether to treat asymptomatic bacteriuria during catheterization or employ prophylactic antibiotics at catheter removal to prevent symptomatic UTIs^3^.

National guidelines further complicate the matter, with divergent recommendations regarding antibiotic prophylaxis at catheter removal. For instance, the American Urological Association^4^ suggests considering prophylaxis for selected patients, while the Infectious Diseases Society of America^3^ and the European Association of Urology^5^ discourage antibiotic use to prevent UTIs. The European Association of Urology guideline also advises against treating asymptomatic catheter-associated bacteriuria.

Given these disparate recommendations and the absence of a unified stance, this study’s primary objective was to conduct a comprehensive systematic literature review and meta-analysis. Through meticulous analysis and synthesis of existing evidence, we aimed to critically assess the efficacy of antibiotic prophylaxis at the time of Foley catheter removal in patients who have undergone radical prostatectomy.

## Methods

### Search strategy

This systematic review and meta-analysis were conducted and reported in accordance with the Cochrane Collaboration Handbook for Systematic Review of interventions and the Preferred reporting Items for Systematic Reviews and Meta-Analysis Statement guidelines^6^.

We searched on MEDLINE, Latin American and Caribbean Health Sciences Literature, and ISI/Web of Science platforms from its inception to June 2023 for retrospective and clinical studies on patients who underwent radical prostatectomy. Our search strategy was based on the use of the descriptors “radical prostatectomy” and “urinary tract infection.”

The references from all included studies, previous systematic reviews and meta-analyses were also searched manually for any additional studies. The prospective meta-analysis protocol was registered on PROSPERO under protocol CRD42023420820.

### Eligibility criteria for study selection

We included:

- Randomized trials (RCTs) or non-randomized cohorts (non-RCTs);
- Studies that assessed the efficacy of antibiotic prophylaxis regarding the Foley catheter removal in patients who underwent radical prostatectomy.

In addition, studies were included only if they reported any of the clinical outcomes of interest.

We excluded studies with patients not submitted to radical prostatectomy; and patients submitted to radical prostatectomy with a cystostomy catheter instead of a urethral catheter.

### Endpoints and subgroup analysis

The primary endpoint assessed was the risk of urinary infection after indwelling urinary catheter removal after radical prostatectomy. Secondarily, we analyzed the presence or absence of bacteriuria in these patients.

### Screening

After deduplication using Endnote online™ 20^7^, a reference management software from Clarivate, Philadelphia, United States of America, two independent researchers (LS and BP) screened the studies by title and abstract, and disagreements were solved by a third one (JA). Following this process, full text screening was performed.

### Data extraction and quality assessment

Two authors (LS and BP) independently extracted the data based on a predefined protocol and disagreements were solved by a third (JA). Our primary outcome, risk of urinary infection, was extracted from all studies here included^8^^–^ 10, while the bacteriuria rate could only be assessed in Fang et al.^8^ and Berrondo et al.^9^ trials.

Risk of bias was assessed in randomized studies using version 2 of the Cochrane Risk of Bias assessment tool (RoB 2)^11^. The non-randomized study was assessed with the Risk of Bias in Non-randomized Studies – of Interventions tool (ROBINS-I)^12^. Two independent authors completed the risk of bias assessment (LS and BP). Disagreements were resolved through a consensus after discussing reasons for discrepancy.

### Statistical analysis

Dichotomous data are presented as odds ratio (OR) with 95% confidence interval (95%CI) in an attempt to a better risk comprehension. Pooled estimates were calculated with the random-effects model, considering that the patients came from different populations. Review Manager 5.4^13^ (Cochrane Center, The Cochrane Collaboration, Denmark) was used for statistical analysis.

## Results

### Study selection and characteristics

**Figure 1:**
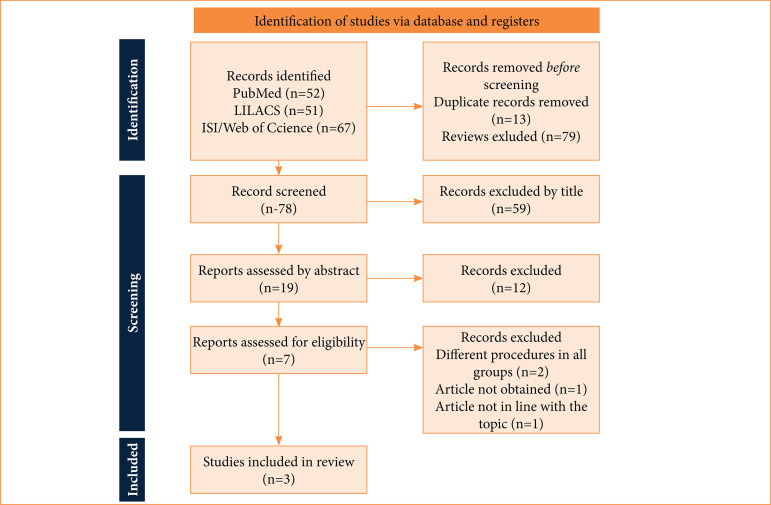
Preferred reporting Items for Systematic Reviews and Meta-Analysis diagram with the results found in the research.

Our search retrieved 170 articles, of which three were included (Fig. 1). Table 1 describes the baseline characteristics of included studies, which were Fang et al.^8^, Berrondo et al.^9^, and Pinochet et al.^10^.

LILACS: Latin American and Caribbean Health Sciences Literature. Source: elaborated by the authors.

**Table 1:** Baseline characteristics of the included studies#.

Study	Type of study	Patients enrolled (N), antibiotic/control	Mean age (years), antibiotic/control	Mean BMI (Kg/m^2^), antibiotic/control	Mean PSA (ng/dL), antibiotic/control	UTI (N), antibiotic/control	Positive urine culture (N), antibiotic/control	Antibiotic used and dose	Catheter duration (days), antibiotic/control	Postoperative complications (Clavien-Dindo), antibiotic/control	UTI definition
Fang et al.^8^	Randomized prospective trial	80/80	65* ± NS/66* ± NS	NS/NS	1.64* ± NS/1.76* ± NS	3/2	7/9	Oral ciprofloxacin, 500 mg, once daily, for seven days	8* ± 3.5/9* ± 2.7	3 patients grade I (3.75%), 2 grade III (2.5%)/3 patients grade I (3.8%), 2 grade III (1.3%)	Fever associated with urinary symptoms
Berrondo et al.^9^	Randomized prospective trial	83/84	62.49 ± 6.86/62.98 ± 6.82	28.95 ± 5.06/29.41 ± 5.30	11.03 ± 23.31/7.77 ± 5.36	3/5	26/56	Oral ciprofloxacin, 500 mg, twice daily, for one day	9.82 ± 1.77/10.08 ± 1.81	8 patients grade I (9.6%)/12 patients grade I (14.3%)	Positive urine culture with at least one organism exceeding 100,000 CFU/mL, coupled with the presence of at least one symptom or sign compatible with UTI
Pinochet et al.^10^	Non-randomized retrospective trial	261/452	60* ± NS/60* ± NS	28* ± NS/27* ± NS	NS/NS	8/33	NS/NS	Oral ciprofloxacin, 500 mg, twice daily, for three days	11 ± NS/7 ± NS	52 patients grade I (20%), 16 grade II (6%), 32 grade III (12%), 1 grade V (0.4%)/91 patients grade I (20%), 34 grade II (8%), 61 grade III (13%)	Patients with lower urinary tract symptoms suggestive of UTI (e.g., burning sensation, increased frequency, and urgency)

# The continuous variables were represented by mean ± standard deviation;

BMI: body mass index; UTI: urinary tract infection; PSA: prostate specific antigen; NS: non specified;

* median

Source: elaborated by the authors.

Regarding the design of the included studies, two of them were RCTs, while one was a non-RCT. We included a total of 1,040 patients, 424 of antibiotic prophylaxis, and 616 of the control group. In the Berrondo et al.^9^ trial, the mean age of all patients was 62.7 years old, with a prostate specific antigen (PSA) mean of 9.4 ng/dL. The therapeutic scheme was based on oral ciprofloxacin, 500 mg, twice daily, for one day. The patients of Fang et al.’s^8^ study presented the mean age of 65.5 years old and the average PSA of 1.7 ng/dL. The therapeutic group received oral ciprofloxacin, 500 mg, once daily, for seven days. Finally, Pinochet et al.^10^ trial involved a series of patients with the average age of 60 years old. The PSA levels were not informed. Also, oral ciprofloxacin, 500 mg, twice daily, for three days, was offered for therapeutic group.

### Meta-analysis

No difference was found when assessing bacteriuria (OR = 0.39; 95%CI 0.12–1.24; p = 0.11; I2 = 73%) (Fig. 2). The data of urine culture was available only for 41 patients (5.75%) in the Pinochet et al.^10^ trial, so this study was not included in this statistical analysis.

**Figure 2:** No difference was found regarding the bacteriuria between the patients.

Study or Subgroup	Antibiotic		Control			Odds Ratio	Odds Ratio
	Events	Total	Events	Total	Weight	M-H, Random,95% CI	M-H, Random,95% CI
Berrondo	26	83	56	84	56.0%	0.23 [0.12, 0.44]	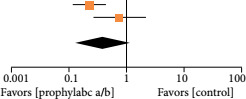
Fang, 2014	7	80	9	80	44.0%	0.76 [0.27, 2.14]	
Total (95% CI)		163		164	100.0%	0.39 [0.12, 1.24]	
Total events	33		65				
Heterogeneity: Tau^2^ = 0.52; Chi^2^ = 3.67, df = 1 (P = 0.06); I^2^ = 73%							
Test for overall effect Z = 1.60 (P = 0.11)							

95%CI: 95% confidence interval. Source: elaborated by the authors.

In terms of UTI, the group who received antibiotic prophylaxis showed superiority over control group (OR = 0.51; 95%CI 0.27–0.98; p = 0.04; I2 = 0%) (Fig. 3).

**Figure 3:** Superiority of antibiotic prophylaxis group over control group in terms of urinary tract infections development.

Study or Subgroup	Antibiotic		Control			Odds Ratio	Odds Ratio
	Events	Total	Events	Total	Weight	M-H, Random,95% CI	M-H, Random,95% CI
Berrondo, 2019	3	83	5	84	19.6%	0.59 [0.14, 2.56]	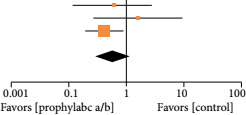
Fang, 2014	3	80	2	80	12.7%	1.52 [0.25, 9.35]	
Pinochet, 2010	8	261	33	452	67.7%	0.40 [0.18, 0.88]	
Total (95% CI)		424		616	100.0%	0.51 [0.27, 0.98]	
Total events	33		65				
Heterogeneity: Tau^2^ = 0.00; Chi^2^ = 1.78, df = 2 (P = 0.41); I^2^ = 0%							
Test for overall effect: Z = 2.02 (P = 0.04)							

95%CI: 95% confidence interval. Source: elaborated by the authors.

### Quality assessment

**Figura 4:**
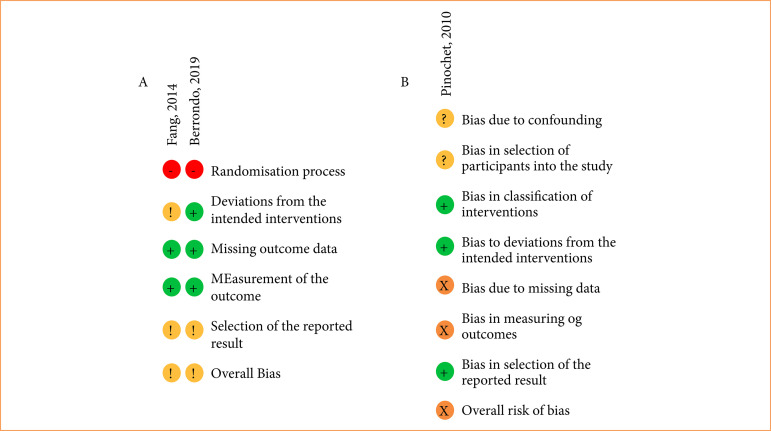
Risk of Bias assessment. **(a)** Risk of bias RoB 2 tool of the included RCT studies. **(b)** Risk of bias ROBINS-I of the included non-RCT study.

Berrondo et al.^9^ and Fang et al.^8^ trials were assessed by Rob-2 tool, and both presented a moderate risk of bias (Fig. 4a). On the other hand, the ROBINS-I score was used in the Pinochet et al.^10^’s study, which showed an overall serious risk of bias (Fig. 4b). It was due to missing data regarding the urinary culture and a possible bias observed in measurement of outcomes.

low risk bias;  some concerns;  high risk of bias;  low risk bias;  moderate risk of bias;  high risk of bias; RCT: randomized trials; RoB 2: Cochrane Risk of Bias assessment tool; ROBINS-I: Risk of Bias in Non-randomized Studies – of Interventions tool. Source: elaborated by the authors.

## Discussion

According to the guidelines established by the Japanese Urology Association^14^, the use of prophylactic antibiotic is recommended for a 24-hour duration in clean surgeries, which are performed in sterile tissues or tissues that can be decontaminated, in the absence of local infectious and inflammatory processes or major technical failures. In cases of clean-contaminated surgeries, the recommended prophylaxis duration extends to 48 to 72 hours. For surgeries classified as non-clean, particularly those involving proximity to intestinal loops, the guideline suggests antibiotic use for approximately 72 to 96 hours. Notably, these recommendations contrast with those provided by the American Urological Association^4^ and the European Association of Urology^5^. However, there is a growing consensus to reconcile these differing guidelines and implement a single-dose protocol for urological surgeries. This dilemma serves as a focal point for our investigation.

Considering the scope of our meta-analysis, which encompasses three pivotal studies (Table 1), we observed that the group receiving antibiotic prophylaxis experienced fewer UTI events in comparison to the control group. Remarkably, there were no significant differences in bacteriuria incidence among the patients included in these studies.

The decreased rate of UTIs observed in the intervention group can be attributed to the early initiation of antibiotic prophylaxis, as illustrated in Fig. 3. Notably, it is essential to highlight variations in the timing of antibiotic administration among the studies. Specifically, Pinochet et al.^10^ administered antibiotics a day before catheter removal, while Fang et al.^8^ and Berrondo et al.^9^ initiated antibiotic therapy on the day of catheter removal.

Regarding the assessment of bacteriuria (as depicted in Fig. 2), our meta-analysis focused exclusively on the trials conducted by Fang et al.^8^ and Berrondo et al.^9^. This selection was made due to a significant methodological difference in the Pinochet et al.^10^’s study, once urine culture was performed solely for patients with prior suspicion of UTIs. This selective approach invalidates its inclusion in the comparative analysis of bacteriuria rates. It’s important to highlight the significant heterogeneity observed in this parameter, with an I^2^ value of 73%. Nevertheless, caution must be exercised when interpreting this information due to the limited number of articles, which reduces the reliability of this parameter. Additionally, the choice of a random-effects model was warranted given the diverse nature of the populations under assessment.

The Clavien-Dindo classification system^15^ was employed to evaluate the postoperative complications across the included studies. In Berrondo et al.^9^ cohort, the intervention group exhibited eight patients classified as grade I (9.6%), while the control group had 12 patients with the same grade I (14.3%). In Fang et al.^8^ cohort, the intervention group had three patients categorized as grade I (3.75%) and two as grade III (2.5%), whereas the control group had three patients classified as grade I (3.8%) and two as grade III (1.3%). Lastly, Pinochet et al.^10^ cohort displayed notable variations in complication grading. In the intervention branch, there were 52 patients with grade I complications (20%), 16 with grade II (6%), 32 with grade III (12%), and one with grade V (0.4%). There was no data about grade IV complications in any of included studies. Conversely, in the control group, there were 91 patients with grade I complications (20%), 34 with grade II (8%), and 61 with grade III (13%). These differences in complication grading among the studies can likely be attributed to the diverse patient populations within each study. Notably, the studies encompassed patients at different stages of prostate cancer, indicating varying disease severity and surgical complexities.

In general, the diagnosis of UTI depends on the presence of genitourinary symptoms such as dysuria, suprapubic pain, hematuria, and a marked increase in urgency or frequency, often coupled with a confirmed positive urine culture^16^^–^ 18. However, it’s important to know that consensus on UTI diagnosis can vary considerably based on factors such as patient age, institutionalization status, and the presence of indwelling urinary catheters^16^.

This lack of a gold standard definition for UTI has contributed to a situation in which there is a prevalent tendency to overprescribe antibiotics in cases in which UTI is suspected. The overuse of antibiotics has far-reaching consequences, with the development of multidrug-resistant pathogens being one of the most pressing concerns. Our study reflects this complex reality by highlighting the varying definitions employed by different researchers. For instance, Berrondo et al.^9^ adopted the symptomatic UTI definition proposed by the Centers for Disease Control, which encompasses a positive urine culture with at least one organism exceeding 100,000 CFU/mL, coupled with the presence of at least one symptom or sign compatible with UTI, such as dysuria, urinary frequency, urinary retention, fever, suprapubic or abdominal pain, or hematuria^19^. Fang et al.^8^, on the other hand, defined UTI patients as those presenting with fever associated with urinary symptoms, while bacteriuria was defined as a urine culture yielding at least 105 CFU/mL. Pinochet et al.^10^’s trial distinguished patients with lower urinary tract symptoms suggestive of UTI (e.g., burning sensation, increased frequency, and urgency) from those presenting with fever, hematuria, or abdominal pain.

The effectiveness of antibiotic prophylaxis has been a subject of investigation in various studies. Bootsma et al.^20^ conducted a comprehensive systematic review in 2008, focusing on antibiotic prophylaxis in various urologic procedures. Their findings revealed a moderate to high level of evidence supporting the use of antibiotic prophylaxis in specific urologic interventions, such as transurethral resection of the prostate, prostate biopsy, ureterorenoscopy, and percutaneous nephrolithotomy. In contrast, this prophylaxis measure was not recommended for other urological procedures, including cystoscopy, urodynamic investigation, transurethral resection of bladder tumor, and extracorporeal shock-wave lithotripsy. The rationale behind this distinction was the lack of well-executed studies investigating the true necessity of antibiotic prophylaxis for these procedures. These insights suggest that antibiotic prophylaxis is well-founded in certain surgical contexts while remaining a subject of ongoing study and evaluation in others.

Moreover, the study conducted by Shin et al.^21^ assessed the antibiotic prophylaxis following RP, specifically comparing the incidence of perioperative infections. This trial involved two distinct patient groups (153/160 patients), with the first group receiving second-generation cephalosporins for less than two days, while the second group received the same drug for more than two days. The results were promising as they demonstrated a significantly higher incidence of postoperative urinary tract infection in the first group (56.9%) compared to the second group (0.6%), which received extended antimicrobial prophylaxis. This finding raises another critical question for future studies: determining the optimal duration of prophylaxis that patients should undergo, a factor that could significantly influence infection outcomes.

Furthermore, the support for antibiotic prophylaxis finds reinforcement in a multicenter study conducted by Togo et al.^22^. This extensive study encompassed 4,677 patients who underwent various urological surgeries and received antimicrobial prophylaxis. The results reported in this study were notably encouraging, as they demonstrated the effectiveness and practicality of a single-dose antibiotic prophylaxis regimen in preventing perioperative infections in the context of urological surgery.

It is noteworthy that the establishment of guidelines for antimicrobial prophylaxis during indwelling urinary catheter removal remains a complex and evolving challenge, as elucidated in the previous discussion^3^^–^ 5 ^,^ 14. Besides, a significant proportion of research studies primarily focuses on prophylaxis administration during the anesthetic induction phase. In contemporary literature, a discernible shift towards supporting prophylaxis emerges, particularly for select patient groups; however, the precise criteria for this selection are still evolving. These targeted prophylaxis recommendations likely pertain to high-risk patient cohorts, which may include individuals of advanced age, those with substantial comorbidities, or those with long-term catheterization history^23^^–^ 26. The strategic restriction of prophylaxis to these specific populations aims to curb the potential emergence of multi-drug resistant uropathogens, a concerning consequence linked to the indiscriminate use of antibiotics. This not only compromises patient outcomes, exposing them to side effects and financial burdens, but also poses a grave threat to public health by limiting our antibiotic arsenal, underscoring the critical importance of addressing antibiotic overprescription.

Our study presents some limitations that warrant consideration. Firstly, the scarcity of literature on this specific topic has resulted in a relatively small sample size, which in turn has constrained the depth and robustness of our analysis, potentially restricting the generalizability of our findings. Secondly, associated with the paucity of existing literature on this topic, the three studies included exhibited variations in study design, ranging from RCTs to non-RCT. This diversity, to some extent, may limit the generalizability of the meta-analysis results. Thirdly, the diagnostic criteria for UTI varied slightly among the included studies, making direct comparisons challenging, introducing some uncertainty into our analysis, and demonstrating the pivotal need for creating standardized criteria for UTI in studies on the subject. Fourthly, we did not account for local antimicrobial resistance patterns, which can significantly impact the infection rates. This omission is important, as it could have influenced the observed outcomes.

The research conducted has some important questions unanswered. One notable issue concerns the formal analysis of complication between the intervention and control group. Additionally, we were unable to definitively determine whether patients treated empirically for UTIs actually had UTIs as the cause of their symptoms. This uncertainty underscores the need for further investigation to comprehensively assess the implications of antibiotic prophylaxis in patients after RP, particularly in preventing the clinical manifestation of UTIs.

Considering the slight superiority of antibiotic prophylaxis observed in our study, we believe that further research in this field is essential to provide stronger evidence in favor of one technique over the other, or even to determine if these techniques yield similar results. This meta-analysis represents the first attempt to compare the efficacy of antibiotic prophylaxis following RP, and, with additional studies, we may obtain more robust evidence that could potentially lead to updates in clinical guidelines.

## Conclusion

Considering our thorough examination of the literature and the meticulous meta-analysis conducted to evaluate the potential advantages of administering antibiotic prophylaxis upon catheter removal following radical prostatectomy, a clear trend emerges. Our analysis indicates a distinct advantage in favor of the prophylactic antibiotic group when it comes to UTI prevention. It’s noteworthy that, while the data did not reveal a statistically significant difference in the presence of bacteriuria, the overall trend leans towards favoring prophylactic antibiotics.

## Data Availability

Available upon request.
